# Key Updates to the 2024 ESC Hypertension Guidelines and Future Perspectives

**DOI:** 10.3390/medicina61020193

**Published:** 2025-01-23

**Authors:** Alexandru Burlacu, Masanari Kuwabara, Crischentian Brinza, Mehmet Kanbay

**Affiliations:** 1 Faculty of Medicine, University of Medicine and Pharmacy “Grigore T Popa”, 700115 Iasi, Romania; 2 Institute of Cardiovascular Diseases “Prof. Dr. George I.M. Georgescu”, 700503 Iasi, Romania; 3 Division of Public Health, Center for Community Medicine, Jichi Medical University, Shimotsuke 329-0431, Japan; kuwamasa728@gmail.com; 4 Division of Cardiovascular Medicine, Department of Medicine, Jichi Medical University, Shimotsuke 329-0431, Japan; 5 Department of Medicine, Division of Nephrology, School of Medicine, Koc University, 34450 Istanbul, Turkey; mkanbay@ku.edu.tr

**Keywords:** hypertension, blood pressure management, cardiovascular risk, guidelines, personalized treatment

## Abstract

Hypertension remains a critical global health challenge, significantly contributing to cardiovascular morbidity and mortality despite advancements in treatment. The 2024 ESC hypertension guidelines address persistent gaps in hypertension management by emphasizing comprehensive strategies encompassing early detection, socioeconomic barriers, lifestyle interventions, and personalized care. Enhanced screening protocols, including home and ambulatory blood pressure monitoring, aim for accurate diagnosis and risk stratification. Lifestyle recommendations now prioritize reducing sodium intake, increasing potassium consumption, and integrating tailored exercise regimens. Pharmacological updates advocate for single-pill combinations and stringent BP targets (<130/80 mmHg), emphasizing the benefits of sodium-glucose cotransporter-2 inhibitors for specific comorbidities. Minimally invasive therapies like renal denervation are explored for resistant hypertension, while digital tools such as telehealth and mobile applications enhance patient engagement and adherence. This multifaceted, patient-centered approach provides a roadmap for optimizing BP control, reducing cardiovascular risks, and addressing the complexities of hypertension in diverse populations.

## 1. Introduction

Arterial hypertension remains a leading global health concern with a substantial impact on cardiovascular (CV) morbidity and mortality, even as we enter 2025. Despite decades of research and clinical advances, many patients still struggle to reach optimal blood pressure (BP) control, and complications from uncontrolled hypertension continue to be alarmingly prevalent.

Hypertension affects over 1.4 billion adults worldwide, with the highest prevalence observed in low- and middle-income countries (LMICs), where nearly three-quarters of cases are reported [1]. Epidemiological data show that the prevalence of hypertension has increased in LMICs over the past two decades, from 24% in 2000 to over 31% in 2010, largely driven by population aging, urbanization, and lifestyle changes. Hypertension control remains suboptimal globally, with fewer than one in five individuals achieving adequate blood pressure control [2]. This gap in management contributes to a significant public health burden, as hypertension is directly linked to increased risks of stroke, heart attack, heart failure, and chronic kidney disease [1,3,4].

**Figure 1:**
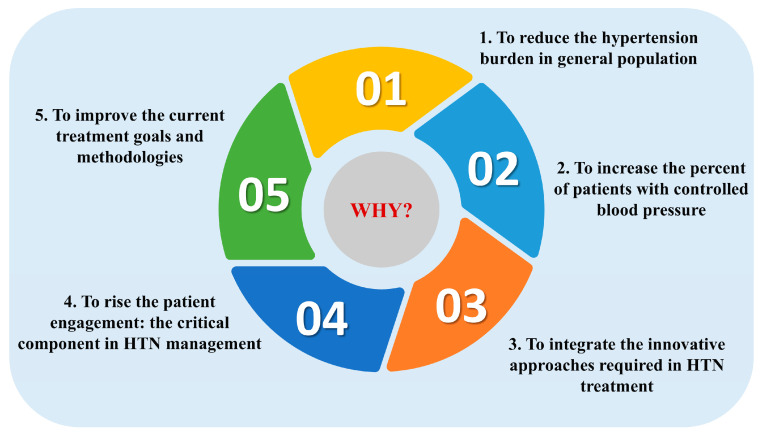
The five answers to the question, “WHY was it already necessary to upgrade the hypertension guidelines?”.

Before exploring what is new, it is crucial to understand why we need new approaches. Where do we stand in 2025? This question prompts us to examine not only the limitations of current approaches but also the unmet needs in prevention, adherence, and personalized care (Figure 1). Only by addressing these gaps can we achieve more effective and sustainable management for those most at risk.

1. Why does hypertension continue to be a major public health issue?

Despite the availability of treatments and awareness campaigns, hypertension still affects a vast portion of the global population. Effective BP control remains challenging, and the long-term consequences of uncontrolled hypertension continue to be severe, contributing to millions of deaths each year [5].

2. Why do many individuals struggle to control their blood pressure?

Non-adherence to treatment, medication side effects, and the lack of personalized management are key factors that hinder effective BP control. Many patients discontinue treatment or fail to monitor their BP regularly, leading to unmonitored fluctuations and increased risk of complications [5,6,7].

3. Why is there a need for innovative approaches in hypertension management?

Traditional management approaches often fail to address the complexities of hypertension, particularly in diverse populations with varied risk factors and comorbidities. Innovative approaches—such as digital health tools, risk stratification, and personalized treatment plans—could address these gaps by providing more tailored and accessible care options [5,8].

4. Why is patient engagement a critical component in hypertension management?

Long-term control of BP requires active patient engagement and education. Without understanding the importance of lifestyle changes and consistent medication use, patients are less likely to adhere to their treatment regimens. Enhanced education and engagement strategies are needed to empower patients to take control of their health [7,9].

5. Why should we reassess our current treatment goals and methodologies?

As research advances, new insights into optimal BP targets and effective interventions emerge. A reassessment of current methodologies allows for integrating evidence-based practices, potentially improving patient outcomes by adapting treatment goals to fit individual needs and risk profiles better [5,10].

Having examined the critical reasons for advancing hypertension management, the next question becomes, in what directions should we focus our efforts to achieve meaningful progress? Addressing this challenge requires a multi-faceted approach that incorporates both innovative practices and fundamental care strategies. Here are five key directions to consider:

1. Implement enhanced screening and early detection.

Regular BP monitoring in both clinical and non-clinical settings, especially among high-risk groups, is crucial for early diagnosis and prompt intervention [11].

2. Address social and economic barriers.

Socioeconomic disparities impact hypertension outcomes, making it essential to prioritize affordable treatment options and policies that improve healthcare access across different populations [11,12].

3. Strengthen the focus on lifestyle interventions.

Sustainable lifestyle modifications, including dietary adjustments, physical activity, and reduced sodium intake, should be integral to hypertension management, complementing medical treatment to achieve optimal BP control [11,13].

4. Leverage technology to enhance patient engagement.

Digital tools, such as mobile applications for BP tracking and telemedicine, provide valuable support in educating and empowering patients, enhancing adherence and consistent monitoring. Furthermore, advancements in interventional techniques such as renal denervation for resistant hypertension and other device-based therapies offer promising alternatives for cases where medication alone may be insufficient [11].

5. Develop personalized treatment approaches.

Adapting treatments based on individual risk factors, comorbidities, and patient preferences can significantly improve adherence and outcomes [11].

Together, these directions highlight a comprehensive, patient-centered strategy, emphasizing both preventive measures and personalized care to improve BP control and reduce the CV risks associated with hypertension on a global scale.

After addressing the critical reasons that justify the endeavor (the “5 Whys”) and identifying strategic directions for improvement (the “5 Wheres”), it becomes imperative to examine the latest evidence-based updates in hypertension management.

In the following section, we present the most recent recommendations highlighting significant advancements in both clinical practice and patient care. These updates present not merely incremental changes but represent a shift toward more refined, patient-centered, and scientifically grounded approaches. By integrating new insights from recent research, these recommendations aim to enhance BP control, reduce hypertension-related complications, and address unmet needs in prevention, monitoring, and adherence. This forward-looking perspective provides healthcare professionals with the latest tools and guidelines for adapting their practice to align with modern clinical standards, ultimately supporting more effective and sustainable hypertension management strategies (Table 1).

1. Screening for hypertension.

The ESC 2024 guidelines emphasize enhanced screening protocols, particularly targeting high-risk populations. Systematic screening for primary aldosteronism in all hypertensive adults is now recommended to address its prevalence and significant impact on treatment outcomes (class IIa indication, level of evidence B) [11].

A crucial addition to these guidelines is the emphasis on using validated and calibrated devices for BP measurement. Correct measurement techniques should be standardized, and a consistent approach should be applied for each patient to ensure accuracy. The guidelines underscore the importance of automated and out-of-office measurements, such as ambulatory BP monitoring (ABPM) and home BP monitoring (HBPM), to reliably detect white-coat and masked hypertension [11,14].

The guidelines recommend opportunistic screening for elevated BP and hypertension. For adults under 40 years, screening should occur at least every 3 years, while those aged 40 years and older should undergo annual screening. For individuals with elevated BP who do not meet risk thresholds for treatment, a repeat BP measurement and risk assessment within one year are advised. The use of systematic screening, self-screening, and non-physician screening is encouraged where feasible within healthcare systems [11].

Accurate diagnosis of hypertension is essential for effective management, and while clinic-based blood pressure measurements are commonly used, they may be influenced by factors such as white-coat hypertension, leading to misdiagnosis [15,16]. ABPM offers a more precise assessment by measuring blood pressure at regular intervals over 24 h, capturing variations throughout daily activities and sleep. Despite the initial costs associated with ABPM devices, the long-term benefits and savings due to more accurate diagnoses and appropriate treatments make ABPM a cost-effective strategy for hypertension diagnosis, particularly in settings where resource constraints necessitate efficient allocation of healthcare funds [15,16].

HBPM is highlighted as an important out-of-office approach where patients measure their BP using validated upper-arm oscillometric cuff devices. To ensure consistency, patients should follow the same preparation steps as in clinics. Two measurements should be taken per session, 1–2 min apart, twice daily (morning and evening) for a minimum of 3 days and up to 7 days [11].

Furthermore, special considerations are made for specific patient populations. For individuals with atrial fibrillation, since most automated oscillometric devices are not validated, BP should be measured using manual auscultatory methods wherever possible. The guidelines also recommend assessing orthostatic hypotension during the initial diagnosis of elevated BP or hypertension and periodically when symptoms suggestive of orthostatic changes arise [11].

2. Diagnosis and classification.

The 2024 guidelines redefine the classification of BP to better reflect the continuous and log-linear relationship between BP levels and CV risk [11].

A new category, termed elevated BP, is introduced for office systolic BP of 120–139 mmHg or diastolic BP of 70–89 mmHg. Although pharmacological treatment is not universally recommended for this group, individuals with high global CV risk may benefit from targeted interventions as determined through risk-stratification approaches [11].

The term “non-elevated BP” replaces previous descriptors like “normal BP” or “optimal BP”, defining systolic BP < 120 mmHg and diastolic BP < 70 mmHg. While fewer individuals in this range face elevated CV risk, the guidelines highlight that risk begins to increase even below these thresholds, particularly for women. This approach avoids misinterpretation and reinforces the importance of preventive measures across all BP categories [11].

Hypertension is defined as a confirmed office systolic BP of ≥140 mmHg or diastolic BP of ≥90 mmHg. To establish this diagnosis, confirmation through out-of-office measurements such as HBPM or ABPM is recommended, or alternatively, a repeat office measurement on a subsequent visit. This definition is supported by meta-analyses showing the benefits of BP-lowering therapy in reducing CV risk for patients above these thresholds. The guidelines also highlight that most individuals with BP ≥ 140/90 mmHg are at an elevated CV risk, often with a 10-year risk of ≥10% for major events [11,17]. Maintaining the traditional threshold avoids overdiagnosis and labeling while focusing on clinical utility [11].

The 2024 classification system integrates the latest evidence from trials while maintaining practical thresholds that balance the treatment benefits against the risks of overdiagnosis. It encourages a focus on risk-based management strategies and lifestyle interventions to prevent progression to hypertension [11].

3. Non-pharmacological lifestyle interventions.

The 2024 guidelines place a stronger emphasis on tailored lifestyle interventions as the cornerstone of managing and preventing hypertension. A major update is the specific goal of reducing sodium intake to below 2 g per day, reflecting robust evidence that lower sodium consumption significantly decreases BP [11,18]. In parallel, increasing dietary potassium is recommended, particularly for individuals with low potassium intake, as a complementary measure to counteract the effects of high sodium levels [11,19].

Evidence from randomized controlled trials and population-level studies consistently demonstrates that reducing salt intake has a significant impact on both blood pressure and cardiovascular outcomes [20]. One notable example is the UK salt reduction program, which achieved a 15% reduction in salt intake between 2003 and 2011, resulting in a 2.7 mmHg decrease in systolic blood pressure at the population level. This decline in blood pressure was associated with reductions in mortality from stroke and ischemic heart disease, highlighting the broader cardiovascular benefits of modest dietary changes. The program underscores the potential of public health initiatives to address hypertension by promoting gradual reductions in salt consumption, thereby lowering the risk of cardiovascular disease across entire populations [20].

Physical activity remains a key intervention, with updated recommendations highlighting the benefits of aerobic and isometric exercises. Structured programs tailored to individual fitness levels are encouraged, as clinical trials have shown that combining dynamic activities such as walking or cycling with isometric exercises can optimize BP reduction [11,21,22,23].

Regular aerobic exercise plays a significant role in managing hypertension, with strong evidence supporting a dose-dependent relationship between physical activity and blood pressure reduction [24,25,26]. A recent meta-analysis of randomized trials demonstrated that engaging in 150 min of aerobic exercise per week yields the most substantial reductions in blood pressure, with a mean decrease of 7.23 mmHg in systolic blood pressure and 5.58 mmHg in diastolic blood pressure. These reductions are clinically meaningful, particularly for patients with hypertension, in whom even small decreases in blood pressure can significantly lower cardiovascular risk [24].

Alcohol consumption recommendations have been refined, advocating for stricter limits to mitigate its dose-dependent effect on hypertension. Recommendations also include avoidance of sugar-sweetened soft drinks, reflecting their potential contribution to elevated BP and metabolic disturbances [11,27,28].

The 2024 guidelines also introduce practical strategies to enhance adherence to lifestyle changes. These include patient education programs; the integration of digital health tools for tracking diet and exercise; and multidisciplinary care models involving dietitians, physiotherapists, and psychologists. By addressing barriers to implementation, these strategies aim to promote sustainable behavior changes and maximize the impact of non-pharmacological interventions [11].

Enhancing patient adherence to lifestyle interventions is crucial for effective hypertension management [29]. Incorporating strategies such as community-based programs and patient education initiatives can significantly improve adherence rates. Additionally, educational interventions that include supportive methods like phone calls, message reminders, and reading materials have demonstrated moderate to large effects on adherence to lifestyle modifications and blood pressure control. Implementing these strategies can bridge the gap between recommended lifestyle changes and actual patient behavior, leading to better health outcomes [29].

4. Pharmacological treatment.

The 2024 guidelines present significant advancements in pharmacological management aimed at simplifying treatment regimens and improving adherence. A key recommendation is the use of single-pill combinations as the first-line treatment for most patients. Combining two or more antihypertensive agents in a single pill can reduce pill burden and improve medication adherence, ultimately leading to better BP control [11,30].

An aggressive BP target of <130/80 mmHg is now advocated for most patients, provided it is well tolerated. This reflects robust evidence linking lower BP targets to reduced CV events, particularly in high-risk groups such as those with diabetes, chronic kidney disease, or established CV disease [11,31]. For older patients, the guidelines highlight the importance of individualized treatment strategies, avoiding overaggressive targets in those with frailty or multiple comorbidities [11,32].

New evidence expands on the use of evidence-based drug classes. Renin–angiotensin system inhibitors, calcium channel blockers, and thiazide-like diuretics remain central to therapy. Additionally, sodium-glucose cotransporter-2 (SGLT2) inhibitors are recognized for their benefits in hypertensive patients with chronic kidney disease, diabetes, or heart failure. In patients with chronic kidney disease, SGLT2 inhibitors have received a class I recommendation, level of evidence A. These drugs offer the dual benefits of lowering BP and improving CV and renal outcomes [11].

A systematic review and meta-analysis of randomized, double-blind, placebo-controlled trials demonstrated that SGLT2 inhibitors consistently reduce both systolic and diastolic BP over a 24 h period [33]. The pooled analysis revealed that these agents decrease 24 h systolic BP by an average of −3.76 mmHg (95% CI: −4.23 to −2.34) and diastolic BP by −1.83 mmHg (95% CI: −2.35 to −1.31). These reductions were observed across both daytime and nighttime periods, suggesting a diurnal effect that may contribute to improved cardiovascular outcomes [33].

The ESC 2024 guidelines recognize the growing evidence supporting the use of SGLT2 inhibitors in hypertensive patients with coexisting conditions such as chronic kidney disease, type 2 diabetes mellitus, and heart failure. By integrating SGLT2 inhibitors into hypertension management, clinicians can achieve the dual benefits of BP control and cardiovascular risk reduction, particularly in high-risk populations.

Addressing medication adherence is a central objective in the 2024 official guidelines. Recommendations include regular follow-ups, patient education, and the use of digital tools to monitor adherence and adjust therapy when necessary. The involvement of multidisciplinary teams, including pharmacists, is encouraged to support patients and optimize treatment outcomes [11].

5. Minimally invasive and device-based interventions.

The 2024 guidelines provide updated insights into the role of minimally invasive and device-based treatments for hypertension, reflecting the growing evidence base supporting their use in specific contexts. These interventions are primarily considered for patients with resistant hypertension who do not achieve target BP levels despite optimal pharmacological treatment and lifestyle modifications [11].

Renal denervation is recognized as a potential adjunctive therapy in the guidelines. Recent randomized trials demonstrated its efficacy in lowering BP, particularly in patients with resistant hypertension. According to the guidelines, renal denervation may be considered for patients with uncontrolled BP despite a three-drug combination therapy, including a thiazide or thiazide-like diuretic, provided the procedure is performed at a medium-to-high-volume center. This option requires that patients express a preference for the procedure following a shared risk–benefit discussion and a thorough multidisciplinary assessment (class IIb, level of evidence B) [11].

Additionally, renal denervation may be an option for patients with increased CV risk and uncontrolled hypertension on fewer than three antihypertensive medications, with the same conditions for shared decision making and multidisciplinary evaluation (class IIb, level of evidence A) [11].

Renal denervation is not recommended as a first-line intervention for BP lowering due to the lack of adequately powered outcome trials demonstrating its safety and CV benefits. Furthermore, it is contraindicated in patients with moderate-to-severe renal impairment (estimated glomerular filtration rate, eGFR, <40 mL/min/1.73 m^2^) or those with secondary causes of hypertension until more evidence becomes available [11].

Recent randomized trials and long-term observational studies have confirmed the efficacy of RDN in reducing blood pressure levels over time [34]. In a 10-year follow-up study of patients who underwent radiofrequency renal denervation, 24 h ambulatory systolic blood pressure decreased by an average of 16 ± 17 mmHg (*p* < 0.001), while office systolic blood pressure was reduced by 14 ± 23 mmHg (*p* = 0.001). These reductions were sustained over the entire follow-up period, demonstrating the long-term effectiveness of the procedure. Despite the sustained efficacy, some challenges remain. The study observed that the number of antihypertensive medications required by patients remained relatively unchanged over 10 years (from 4.9 ± 1.4 to 4.5 ± 1.2 medications, *p* = 0.087), indicating that RDN is not a replacement for pharmacological treatment, but rather, a complementary therapy [34].

While initial trials reported potential benefits for baroreceptor activation therapy, the evidence remains preliminary, and its application is currently limited to clinical trial settings or highly specialized centers with expertise in managing complex hypertension cases [11].

Patient selection remains a critical factor to optimizing the outcomes of RDN [35,36,37]. Recent evidence suggests that higher baseline heart rate and lower pulse wave velocity are among the most reliable predictors of significant blood pressure reductions after the procedure [35]. Specifically, patients with a higher baseline heart rate demonstrated a 24 h systolic BP reduction of −4.05 mmHg (95% CI: −7.33 to −0.77) and daytime systolic BP reduction of −5.99 mmHg (95% CI: −9.49 to −2.50). In contrast, individuals with a lower pulse wave velocity experienced a 24 h systolic BP reduction of −7.20 mmHg (95% CI: −9.79 to −4.62) and daytime systolic BP reduction of −9.09 mmHg (95% CI: −11.63 to −6.55), indicating that vascular stiffness plays a significant role in determining the efficacy of RDN [35].

Candidates should undergo comprehensive evaluation at specialized hypertension centers to confirm true treatment resistance and exclude secondary causes of hypertension. These interventions should be integrated within a multidisciplinary care framework, ensuring they complement rather than replace conventional therapies [11].

6. Integration of digital tools in hypertension management.

The 2024 guidelines introduce a forward-looking perspective on the use of digital tools for hypertension management, emphasizing their potential to enhance patient care through improved monitoring, adherence, and individualized treatment strategies. These tools, ranging from mobile applications to wearable devices, have become increasingly effective in hypertension care [11].

One significant advancement is the recommendation for digital platforms that support HBPM. These platforms allow patients to record and share their BP readings with healthcare providers in real time, fostering a collaborative approach to care. By integrating validated mobile applications with HBPM devices, patients can track trends over time, receive automated reminders for measurements, and access educational resources tailored to their condition. Such features are designed to improve adherence to self-monitoring protocols [11].

Telehealth services have been given a more prominent role in the 2024 guidelines, reflecting their rapid adoption in recent years. Virtual consultations enable patients to discuss their hypertension management with healthcare providers without the need for in-person visits, reducing barriers such as time and travel constraints. Telehealth platforms often incorporate secure messaging, video calls, and integrated monitoring data, creating an environment for continuous care [11].

One study reported a mean reduction of 14.1 mmHg in systolic BP (*p* < 0.001) over six months in a cohort of predominantly low-income patients using a home BP telemonitoring program [38]. This intervention involved regular remote BP reviews and protocol-based medication adjustments by a clinical pharmacist, highlighting the feasibility and effectiveness of such programs in real-world settings [38].

The Hyperlink study demonstrated that combining home BP monitoring with pharmacist-led co-management led to substantial reductions in BP over a 12-month period [39]. The intervention included lifestyle counseling, medication review, and systematic treatment adjustments, which were made based on transmitted BP data [39].

In addition to improving access to care, telemonitoring programs enhance patient engagement and self-management [40]. Patients are empowered to take an active role in their treatment by performing regular BP measurements at home, which can lead to improved adherence to antihypertensive therapy. However, accurate measurements require proper training on technique. McGrath et al. emphasized the importance of ensuring that patients are taught correct BP measurement practices during video consultations or in-person visits before initiating telemonitoring programs [40]. This training step is essential to avoid inaccuracies caused by improper cuff positioning or other common errors [40].

However, digital tools also present important ethical and regulatory challenges that must be addressed to ensure safe and equitable implementation [41,42,43]. A key ethical concern involves data privacy and security, as digital tools collect and store sensitive health information. Ensuring that personal data are securely managed and protected from breaches is essential to maintaining patient trust and compliance [41]. Another challenge is equity of access, as digital tools may unintentionally widen healthcare disparities if certain populations lack access to the necessary technology or digital literacy [42]. Addressing this issue requires policies that promote equal access to and education on using digital health solutions effectively. The regulatory approval processes for digital health technologies remain complex, often delaying the implementation of beneficial innovations. Policymakers must create frameworks that balance the need for safety and efficacy with timely access to new technologies [41,43].

7. Socioeconomic barriers and global applicability of guidelines.

The implementation of the 2024 ESC hypertension guidelines faces significant challenges in LMICs, where healthcare disparities remain profound. Despite the growing global burden of hypertension, particularly in LMICs, these regions often lack the necessary resources to adhere to evidence-based recommendations. Many low-resource settings face a critical shortage of trained healthcare professionals, limiting the capacity for early detection, diagnosis, and ongoing monitoring of hypertension [44,45].

Studies have shown that over 90% of the global burden of cardiovascular disease is concentrated in LMICs, yet these countries possess less than 10% of the healthcare resources required to address the issue. Compared with high-income countries, hypertension control rates in LMICs are substantially lower, with control rates as low as 5–10% in some African countries versus over 50% in the United States. The affordability of essential antihypertensive medications, access to healthcare providers, and availability of out-of-office monitoring tools remain critical barriers [44,45].

8. Evidence gaps and further research directions.

The 2024 ESC guidelines recognize several critical gaps in evidence that require further investigation to optimize hypertension management. One notable gap is the lack of sex-specific data on the epidemiology, risk factors, and pathophysiology of hypertension. The current research often overlooks differences in hormonal, genetic, and sociocultural factors that influence blood pressure control and cardiovascular outcomes in men and women.

The 2024 ESC guidelines also highlight the need for more research on hypertension management in challenging environments, particularly in LMICs, where unique barriers to care exist. These include climate change, pollution, and pandemics, which have direct and indirect impacts on cardiovascular health and the accessibility of healthcare services.

## 2. Conclusions

In conclusion, addressing hypertension effectively requires a multi-faceted approach that includes improved screening, addressing socioeconomic barriers, and promoting lifestyle modifications such as reduced sodium intake, physical activity, and reduced alcohol consumption. The 2024 guidelines highlight the importance of personalized treatment approaches, leveraging technology like digital tools for patient monitoring and minimally invasive interventions for resistant cases. By integrating these strategies, healthcare providers can enhance BP control and reduce CV risks, ultimately improving the quality of life for individuals affected by hypertension.

## Figures and Tables

**Table 1:** Key updates in hypertension management.

HTN screening	Recommend systematic screening for primary aldosteronism in hypertensive adults.
	Emphasize validated devices and out-of-office BP measurements to improve accuracy.
	Advocate opportunistic screening at regular intervals based on age.
Diagnosis and classification	Introduce new BP categories to refine risk stratification.
	Confirm hypertension diagnosis with out-of-office or repeated office measurements.
	Retain traditional hypertension thresholds to avoid overdiagnosis.
Lifestyle interventions	Promote reducing sodium intake to below 2 g/day.
	Recommend structured aerobic and isometric exercise programs.
	Refine alcohol consumption limits.
	Encourage patient education and digital tools to improve lifestyle adherence.
Pharmacological treatment	Recommend single-pill combinations to improve medication adherence.
	Advocate for BP targets of <130/80 mmHg for most patients.
	Emphasize the use of SGLT2 inhibitors in patients with comorbidities.
	Suggest regular follow-ups and multidisciplinary care for better outcomes.
Minimally invasive interventions	Consider renal denervation as an option for resistant hypertension.
	Require patient selection and shared decision making for invasive therapies.
	Discourage first-line use of RDN due to limited outcome data.
Digital tools in HTN management	Promote telehealth services to reduce care barriers.
	Emphasize training patients to ensure accurate BP measurements.
	Address data privacy and equity challenges in digital tool use.
Socioeconomic barriers	Highlight healthcare disparities in LMICs affecting hypertension management.
	Recommend policies to improve access to medications and monitoring tools.
Evidence gaps	Identify the need for sex-specific data in hypertension management.
	Call for research in challenging environments like LMICs.
	Emphasize the importance of addressing global health disparities.

BP—blood pressure; HTN—hypertension; LMICs—low- and middle-income countries; RDN—renal denervation; SGLT2 inhibitors—sodium-glucose cotransporter-2 inhibitors.

## Data Availability

Not applicable.
